# IL-6-mediated cross-talk between human preadipocytes and ductal carcinoma in situ in breast cancer progression

**DOI:** 10.1186/s13046-018-0867-3

**Published:** 2018-08-22

**Authors:** Hoe Suk Kim, Minji Jung, Sul Ki Choi, Jisu Woo, Yin Ji Piao, Eun Hye Hwang, Hyelim Kim, Seung Ja Kim, Woo Kyung Moon

**Affiliations:** 1 Department of Radiology, Seoul National University Hospital and Seoul National University College of Medicine, 101 Daehak-ro, Jongno-gu, Seoul, 03080 Republic of Korea; 2 Department of Biomedical Sciences, Seoul National University College of Medicine, 103 Daehak-ro, Jongno-gu, Seoul, 03080 Republic of Korea; 3 Department of Radiology, Sheikh Khalifa Specialty Hospital, Ras Al Khaimah, Abu Dhabi, United Arab Emirates

**Keywords:** Ductal carcinoma in situ, Preadipocyte, Interleukin-6, Breast cancer

## Abstract

**Background:**

The function of preadipocytes in the progression of early stage breast cancer has not been fully elucidated at the molecular level. To delineate the role of preadipocytes in breast cancer progression, we investigated the cross-talk between human breast ductal carcinoma in situ (DCIS) cells and preadipocytes with both an in vitro culture and xenograft tumor model.

**Methods:**

GFP or RFP was transduced into human DCIS cell line MCF10DCIS.com cells or preadipocytes using lentivirus. Cell sorter was used to separate pure, viable populations of GFP- or RFP-transduced cells. Cell viability and proliferation was assessed by crystal violet assays and cell migration and invasion capability was assayed by the transwell strategy. Gene and protein levels were measured by western blot, RT-PCR and immunostaining. Adipokines and cytokines were quantified using ELISA. Human tumor xenografts in a nude mice model were used. Ultrasound imaging of tumors was performed to evaluate the therapeutic potential of a IL-6 neutralizing antibody.

**Results:**

In the co-culture system with the MCF10DCIS.com and preadipocytes, MCF10DCIS.com proliferation, migration and invasion were enhanced by preadipocytes. Preadipocytes exhibited in an increased IL-6 secretion and cancer-associated fibroblast markers expression, FSP1 and α-SMC in co-culture with MCF10DCIS.com or in MCF10DCIS.com conditioned media, whereas the adipocyte differentiation capacity was suppressed by co-culture with MCF10DCIS.com. A neutralizing antibody of IL-6 or IL-6R suppressed the promotion of MCF10DCIS.com proliferation and migration by co-culture with preadipocytes. In the xenograft tumor model, the tumor growth of MCF10DCIS.com was enhanced by the co-injection of preadipocytes, and the administration of IL-6 neutralizing antibodies resulted in potent effects on tumor inhibition.

**Conclusions:**

Our findings suggest that IL-6-mediated cross-talk between preadipocytes and breast DCIS cells can promote the progression of early stage breast cancer. Therefore, blocking IL-6 signaling might be a potential therapeutic strategy for breast DCIS characterized by pathological IL-6 overproduction.

**Electronic supplementary material:**

The online version of this article (10.1186/s13046-018-0867-3) contains supplementary material, which is available to authorized users.

## Background

Ductal carcinoma in situ (DCIS), known as intraductal carcinoma, is defined as the proliferation of neoplastic epithelial cells within the breast milk duct. Because DCIS may develop into invasive breast cancer within 5 to 10 years after the initial diagnosis, it is generally recommended that patients with DCIS receive treatment [1]. Based on molecular, epidemiological, and pathological studies [2], DCIS is thought to be an early form of breast cancer, but very little is known about the factors that promote survival in DCIS neoplastic cells.

Identifying the tumor microenvironmental regulators of DCIS progression is critical for determining a predictive molecular signature and for treating patients with DCIS. The most abundant stromal constituent in the breast is adipose tissue. Adipose tissue is an endocrine organ that is primarily composed of heterogeneous cells, including adipocytes and preadipocytes [3]. Recent evidence highlights the adipose tissue surrounding tumors as a key component in breast cancer progression [4–6]. While there is extensive literature on the association of adipose tissue with the invasiveness and the dissemination of breast cancer cells by leptin, hepatocyte growth factor (HGF), collagen VI, interleukin-6 (IL-6) and C-C motif chemokine 5 (CCL5) secreted from adipocytes as well preadipocytes isolated from abdominal and breast adipose tissue [7–13], little is known about the DCIS progression to invasive breast cancer by cross-talk with preadipocytes. A recent study documented that preadipocytes promote the DCIS progression to breast cancer via exosomal signaling [14], implying association of preadipocytes-secreted factors with the development of breast cancer in early stage.

The contribution of factors secreted from human preadipocytes to breast cancer progression at early stage is largely unknown. A better understanding of the cross-talk between preadipocytes and human DCIS cells is essential to clarify targets and strategies for preventing the pro-tumorigenic effect of preadipocytes. Thus, this study aimed to investigate the preadipocyte-derived factors that drive cell migration and proliferation in cultured human DCIS cells and tumor progression in xenograft models and to evaluate if the inhibition of preadipocyte-derived factors can prevent breast cancer development.

## Methods

### Cell culture

Human DCIS cell line MCF10DCIS.com (Asterand, Detroit, MI) which designated as DCIS.com, was cultured in DMEM/F12 supplemented with 5% heat-inactivated horse serum (Invitrogen, Carlsbad, CA), 4 μg/ml insulin (Gibco, Grand Island, NY), 100 ng/ml cholera toxin, 0.5 μg/ml hydrocortisone (Sigma, St. Louis, MO), and 20 ng/ml EGF (Life Technologies, Grand Island, NY). Normal human preadipocytes (hPreAd) isolated from subcutaneous fat from healthy (non-diabetic) donors were purchased from Lonza (Walkersville, MD) and were grown in BulletKit™ media. In this study, DCIS.com/RFP and hPreAd/GFP cells were generated using lentiviruses carrying RFP and GFP, respectively. Briefly, lentiviral vectors containing the RFP or GFP construct were purchased from Addgene (Cambridge, MA) to establish RFP-transduced DCIS.com and GFP-transduced preadipocytes. Lentiviral production and transduction was conducted according to the manufacturer’s instructions (Invitrogen, Carlsbad, CA). At 7 days after lentiviral transduction RFP- or GFP-positive cells were sorted using a FACSCalibur flow cytometer (BD Biosciences, Franklin Lakes, NJ). Cells that stably expressed RFP or GFP were generated for use in in vitro subsequent studies.

### Proliferation assay

To investigate alterations in DCIS.com and hPreAd proliferation in co-culture conditions, a proliferation assay was performed using transwell chambers with 4-μm pore size insert (Costar, Cambridge, MA). For a proliferation assay of DCIS.com in co-culture, the DCIS.com (0.5 × 10^5^ cells/ml) was seeded in the bottom of 24-well transwell plates and the hPreAd in the upper chamber. To test the hPreAd proliferation, the hPreAd (0.5 × 10^5^ cells/ml) were loaded in the bottom of 24-well transwell plates and the DCIS.com in the upper chamber. To evaluate the alterations in DCIS.com proliferation mediated by IL-6, DCIS.com were cultured in medium containing recombinant human IL-6 (50 ng/ml) (R&D Systems, Minneapolis, MN) for 48 h, and transwell co-cultures were performed with hPreAd in the presence or absence of IL-6 neutralizing antibody (NAb) (3 μg/ml) (R&D Systems, Minneapolis, MN) or IL-6R NAb (3 μg/ml) (R&D Systems, Minneapolis, MN) for 48 h. Cells in the lower chamber were stained with 1% crystal violet in methanol/PBS. Crystal violet was then dissolved with a 0.1% SDS solution, and the absorbance was read at 570 nm.

### Migration and invasion assay and confocal microscopy imaging

DCIS.com migration in co-culture with hPreAd was assessed in transwell chambers with an 8-μm pore size insert (Costar, Cambridge, MA). DCIS.com (0.5 × 10^5^ cells/ml) was seeded into the top chamber, and the lower chamber contained hPreAd (0.5 × 10^5^ cells/ml). To evaluate the IL-6-mediated alterations in DCIS.com migration, DCIS.com was cultured in medium containing IL-6 (50 ng/ml) for 48 h, and transwell co-cultures with hPreAd in the presence or absence of IL-6 NAb (3 μg/ml) or IL-6R NAb (3 μg/ml) for 48 h were performed. To assess the invasion assay, DCIS.com was seeded in the upper chamber containing 2% Matrigel (BD Biosciences, Billerica, MA). The migrated cells were stained with 1% crystal violet in methanol/PBS. Crystal violet was then dissolved with a 0.1% SDS solution, and the absorbance was read at 570 nm.

For live imaging of DCIS.com invasion across three-dimensional (3D) Matrigel which mimic the in vivo cellular environment into hPreAd, hPreAd was seeded in an 8-well chamber slide, 2% Matrigel (274-μm-thick) was added, and DCIS.com was seeded onto the Matrigel. Confocal images were obtained using an LSM700 microscope (Zeiss, MJena, Germany), and 3D reconstructions were performed from confocal z-series stacks (20–25 μm) by scanning at 1-μm intervals.

### 3D culture

A layer of growth factor-reduced Matrigel was made for 3D on-top Matrigel cultures [14]. In brief, 24-well culture plates were coated with a thin layer of Matrigel (120 μl) and incubated for 15–30 min at 37 °C to allow the Matrigel to solidify. The mixture of DCIS.com (5–6 × 10^6^ cells/ml) and Matrigel was added onto the pre-coated surface. After hPreAd were cultured for 72 h, the culture supernatants of hPreAd were concentrated and conditioned media of hPreAd were prepared. After incubating for 30 min at 37 °C to allow the Matrigel to gel, the appropriate volume of hPreAd conditioned media containing IL-6 (50 ng/ml), IL-6 NAb (3 μg/ml) or IL-6R NAb (3 μg/ml) was added, and the DCIS.com in the 3D Matrigel culture was grown for an additional 10 days. The formation of spheroids was observed under the fluorescence microscopy (Leica, Wetzlar, Germany).

### RT-PCR and real-time RT-PCR

Total RNA was isolated using TRIzol reagent (Invitrogen, Carlsbad, CA) and was reverse-transcribed using random hexamers and Superscript III reverse transcriptase. cDNAs were synthesized using M-MLV reverse transcriptase (New England Biolabs, Ipswich, MA) and specific primers. IL-6 and Interleukin 6 receptor (IL-6R) mRNA expression levels were measured in single culture and co-culture using the following primer sets (Additional file 1: Table S1). Real-time RT-PCR reactions were run on an ABI PRISM 7900 utilizing a SYBR Green PCR master mix (Applied Biosystems, Foster City, CA). Adipocyte-related genes such as peroxisome proliferator-activated receptor-gamma (PPAR-γ) and lipoprotein lipase (LPL) mRNA expression levels were measured in single culture and co-culture using the specific primer sets (Additional file 1: Table S1). Results were analyzed by the ∆Ct method, which reflects the threshold difference between a target gene and β-actin in each sample.

### Adipocyte differentiation and oil red O staining

Adipocyte differentiation was tested according to previously published protocols [15]. hPreAd were cultured at a density of 5000~ 6000 cells/cm^2^. After reaching confluence, hPreAd was cultured in adipogenic medium containing 0.5 mM isobutyl-methylxanthine, 1 μM dexamethasone, 10 μM insulin, and 100 μM indomethacin for three days, were maintained in medium with 10 μM insulin for 5 days and were subjected to Oil Red O staining to detect cytoplasmic triglycerides. Differentiated adipocytes were rinsed twice with PBS and fixed in 10% formalin at room temperature for 30 min. The working solution of Oil Red O was prepared fresh before staining, and after the plate completely dried, cells were stained for 30 min at room temperature. Images of lipid droplets were acquired using a microscope. For quantitative analysis of Oil Red O contents levels, stained Oil Red O was eluted with isopropanol and quantified by measuring the optical absorbance at 510 nm with microplate reader (Bio-Rad, Hercules, CA).

### Western blotting and immunofluorescence staining

Total cell lysates (50–100 μg) were separated by SDS-PAGE and transferred onto a nitrocellulose membrane. The membrane was incubated with specific primary antibodies overnight at 4 °C, incubated with horseradish peroxidase-conjugated secondary antibody, and visualized with an ECL western blotting detection reagents (Thermo Scientific, Rockford, IL). The following primary antibodies were used in this study: anti-phospho-mTOR, anti-mTOR, anti-phospho-AKT, anti-AKT, anti-phospho-ERK1/2, anti-ERK1/2, anti-phospho-STAT3 and anti-STAT3 antibodies (Cell Signaling Technology, Danvers, MA); anti-phospho-FAK antibody (Abcam, Cambridge, UK); anti-IL-6 and anti-IL-6Rα antibodies (R&D Systems, Minneapolis, MN); anti-FAK and anti-β-actin antibodies (Sigma, St. Louis, MO). The relative intensity of the bands observed by western blotting was analyzed using ImageJ software.

After 72 h single or co-culture in transwell chamber, hPreAd was rinsed with ice-cold PBS and fixed with 4% paraformaldehyde for 10 min at room temperature followed by permeabilization with 0.1% sodium citrate plus 0.1% triton X-100. The cells were subjected to immunofluorescence staining with anti-fibroblast-specific protein 1 (FSP1) (Millipore, Burlington, MA) and anti-α-smooth muscle actin (α-SMC) (Abcam, Cambridge, UK) antibodies for 2 h at room temperature. The cells were then washed with cold PBS three times for 3 min each, and incubated with Alexa 488-labeled secondary antibody at room temperature for 1 h. Cell nuclei were stained with 4′,6-diamidine-2′-phenylindole dihydrochloride (DAPI) (Roche, Berlin, Germany). The cells were examined by fluorescence microscopy (Leica, Wetzlar, Germany).

### Measurement of secreted adipokines and cytokines

DCIS.com and hPreAd were cultured in transwell chamber. After 48 h culture period, the conditioned media in single or co-culture were collected, centrifuged between 5000 to 10,000 rpm for 10 min at 4 °C, filtered using 0.4 μm syringe filter, and stored at − 80 °C. The levels of secreted adipokines and cytokines including adiponectin, leptin, resistin, PAI-1, CCL2, CRP, TGF-β1, TGF-β2, TGF-β3, IL-2, IL-4, IL-5, IL-6, IL-10, IL-12(p70), IL-13, GM-CGS, INF-γ, MMP-1, MMP-2, MMP-3, MMP-9, MMP-12, and MMP-13 were quantified using the Bio-Plex200 multiplex array system according to the recommended protocol (Bio-Rad, Hercules, CA). All samples and standardized solutions were analyzed in at least three independent experiments.

### Xenograft studies

All animal experiments were approved by the Seoul National University Hospital Biomedical Research Institute Animal Care and Use Committee (IACUC 13–0350-C2A0(1)). A total of 16 female BALB/c nude mice were used. Xenografts were established via the subcutaneous injection of 1 × 10^6^ DCIS.com (*n* = 8) or a mixture of 1 × 10^6^ DCIS.com and 1 × 10^6^ hPreAd cells (n = 8) into the flank region of 5-week-old mice. Tumor-bearing mice were randomly assigned to one of four groups: DCIS.com (*n* = 4); DCIS.com+IL-6 NAb (n = 4); DCIS.com+hPreAd (n = 4); and DCIS.com+hPreAd+IL-6 NAb (n = 4). At 7 and 12 day post-cancer cell injection, mice were intravenously injected with IL-6 NAb (2 mg/kg). Tumor volume was measured with digital calipers and US imaging with a modified ellipsoidal formula for volume (volume = 1/2(length×width2)) [16]. At 10 weeks after the subcutaneous injection of DCIS.com, xenograft tumors were removed.

### Ultrasound (US) imaging analysis

US imaging of tumors was performed in B mode using a preclinical Vevo2100 LAZR imaging system (Fujifilm VisualSonics Inc., Toronto, Ontario, Canada) equipped with a 40 MHz linear array transducer.

### Histological analysis

The excised primary tumors were fixed with 4% buffered formalin and embedded in paraffin blocks. Tissues were sectioned into 4-μm thick sections. Paraffin sections were deparaffinized in xylene and rehydrated in a series of graded ethanol and water solutions. Hematoxylin and eosin (H&E) staining was performed to evaluate the change in cell and tissue structure. Histological images of stained tissues were acquired by use of a microscope equipped with a CCD camera (Leica, Wetzlar, Germany).

### Statistical analysis

Statistical analysis was performed using Graph Pad Prism software, and data were assessed by a two-tailed Student’s t-test. The results were considered significant when *P* < 0.05, and data are presented as the means ± standard errors.

## Results

### Co-culture with DCIS.com inhibited adipogenic differentiation of preadipocytes and induced the expression fibroblast marker gene in preadipocytes

**Fig. 1:**
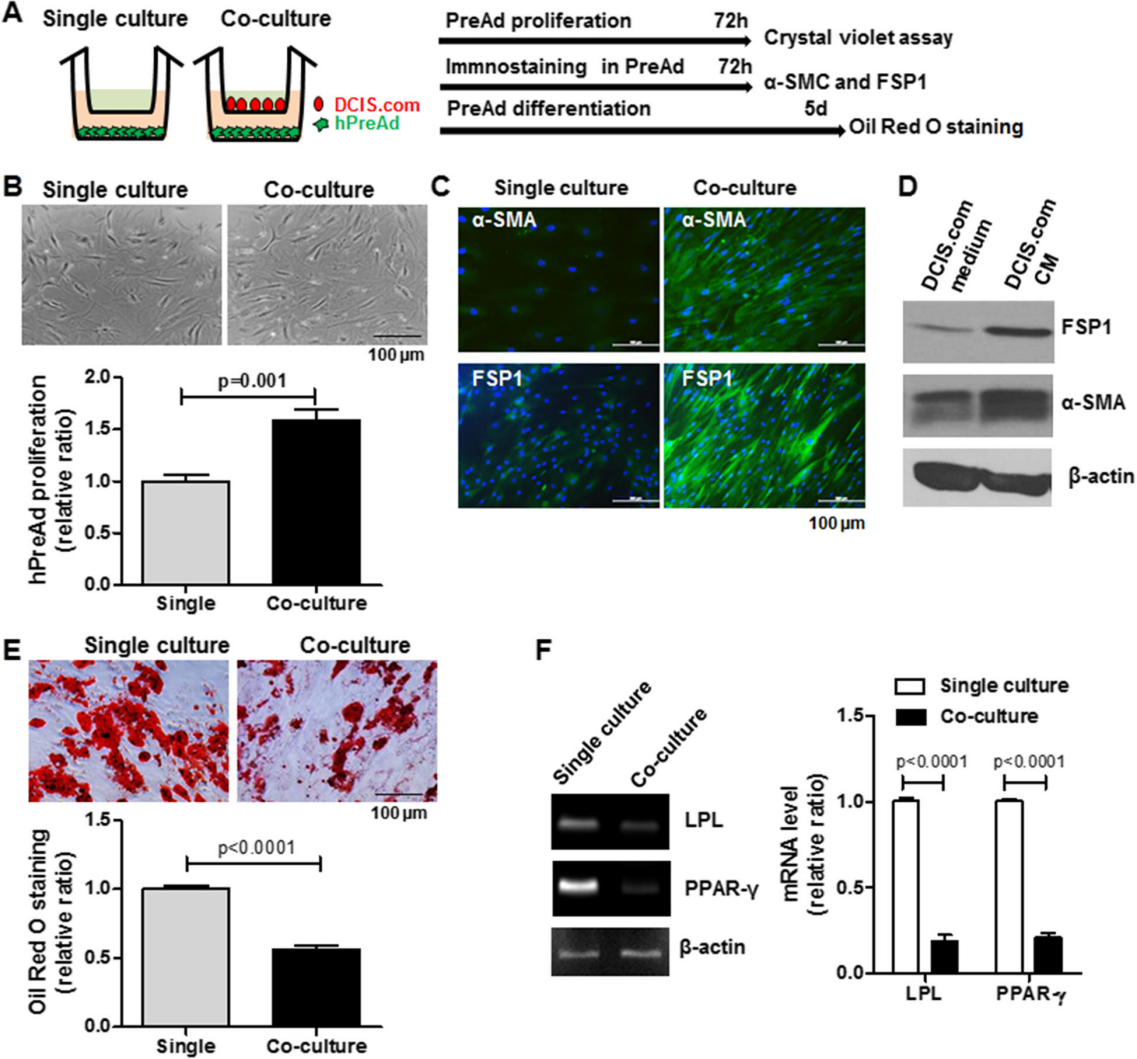
Differentiation ability of human preadipocytes was suppressed by co-culture with DCIS.com, and human preadipocytes appear to revert to a fibroblast phenotype. **a** Experimental scheme illustrating the transwell culture of DCIS.com (DCIS.com) and human preadipocytes (hPreAd). **b** Proliferation analysis of hPreAd in single or co-culture was assessed by crystal violet assays of six independent experiments. **c** Analysis of fibroblast-specific protein 1 (FSP1) and alpha-smooth muscle actin (α-SMC) expression in hPreAd in single or co-culture, as assessed by immunostaining. Green: FSP1 or α-SMC-positive. Blue: nucleus. **d** Analysis of FSP1 and α-SMC expression in hPreAd treated with DCIS.com conditioned medium (CM), as assessed by western blot. **e** Differentiation analysis of hPreAd in single or co-culture was assessed by Oil Red O staining of six independent experiments. **f** Analysis of lipoprotein lipase (LPL) and peroxisome proliferator-activated receptor-gamma (PPAR-γ) mRNA expression in hPreAd in single or co-culture, as assessed by RT-PCR and real-time RT-PCR of three independent experiments. All data are presented as the means ± standard errors

ER, PR and HER2 status in DCIS.com were determined by real-time RT-PCR. DCIS.com belongs to the ER^−^/PR^−^/HER2^−^ and basal-like DCIS subtype as compared with MCF-7 cells (ER^+^/PR^+^ subtype), SK-BR-3 cells (HER2 subtype) and MDA-MB-231 cells (ER^−^/PR^−^/HER2^−^ subtype) (Additional file 1: Figure S1). To investigate the effect of DCIS.com on the proliferation and differentiation of hPreAd, indirect co-culture was performed using transwell assays (Fig. 1a). A 72-h co-culture of hPreAd with DCIS.com resulted in a significant increase in hPreAd proliferation compared with that of the single culture (1.58 ± 0.11 vs 1.0 ± 0.06, *P* = 0.001, Fig. 1b). After a 72-h co-culture of hPreAd with DCIS.com in an in vitro transwell assay, we observed the upregulated expression of two cancer-associated fibroblast markers, FSP1 and α-SMC, and a more elongated cell shape, similar to fibroblast morphology (Fig. 1c). The hPreAd exposed to DCIS.com-conditioned media exhibited an upregulation in FSP1 and α-SMC expression (Fig. 1d). During a 5-day co-culture, the differentiation ability of hPreAd into mature adipocytes was suppressed, and there was a considerable decrease in the amount of lipid droplets (0.56 ± 0.03 vs 1.0 ± 0.07, *P* < 0.0001, Fig. 1e). The expression levels of adipocyte-related genes such as PPAR-γ (1.00 ± 0.02 vs 0.21 ± 0.03, *P* < 0.0001) and LPL (1.00 ± 0.02 vs 0.19 ± 0.03, *P* < 0.0001) were also significantly decreased in co-culture relative to single culture (Fig. 1f).

### The proliferation, migration, and invasion of DCIS.com were enhanced by co-culture with preadipocytes

**Fig. 2:**
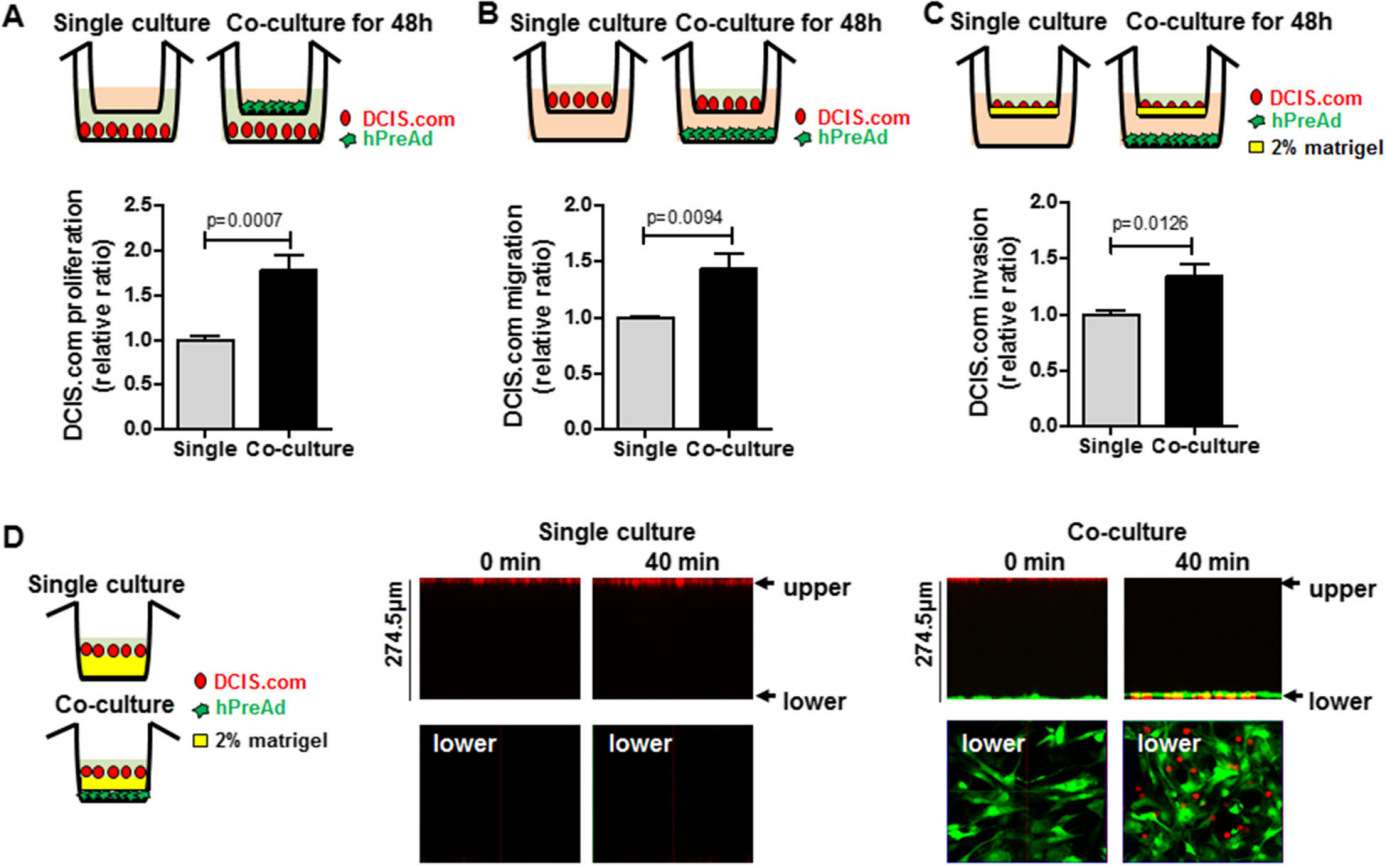
The proliferation, migration, and invasion abilities of DCIS.com were promoted by co-culture with human preadipocytes. Experimental scheme illustrating the transwell culture of DCIS.com (DCIS.com) and human preadipocytes (hPreAd). **a** Proliferation analysis of DCIS.com in single or co-culture, as assessed by crystal violet assay of six independent experiments. **b** Migration analysis of DCIS.com in single or co-culture, as assessed by crystal violet assay of six independent experiments. **c** Invasion analysis of DCIS.com in single or co-culture, as assessed by crystal violet assay of six independent experiments. **d** Fluorescence confocal images of DCIS.com migrating into hPreAd. All data are presented as the means ± standard errors

To investigate the effect of hPreAd-secreted factors on the proliferation, migration, and invasion of DCIS.com, a transwell system was used. Proliferation of DCIS.com was significantly increased in cells co-cultured with hPreAd for 48 h (1.00 ± 0.04 vs 1.78 ± 0.16, *P* = 0.0007, Fig. 2a). A significant increase in the migration of DCIS.com co-cultured with hPreAd was observed compared with that of the single culture (1.44 ± 0.14 vs 1.0 ± 0.01, *P* = 0.009, Fig. 2b). A Matrigel-based cell invasion assay revealed a similar stimulatory effect of hPreAd on DCIS.com invasion (1.35 ± 0.11 vs 1.0 ± 0.03, *P* = 0.0126, Fig. 2c). The Matrigel invasion of RFP-labeled DCIS.com into GFP-labeled hPreAd was reconstructed by 3D real-time confocal images. After 40 min, the invasion of DCIS.com was detected in hPreAd embedded in Matrigel but not in Matrigel without the presence of hPreAd (Fig. 2d). In evaluation of the proliferation and migration of other breast cancer cell lines (basal-like subtype, MDA-MB-231 and HER2+/luminal-like subtype, SUM225) and normal mammary epithelial cell (MCF10A) in co-culture with hPreAd, an increase in proliferation and migration of MDA-MB-231 cell increased but not SUM225 and MCF10A cells (Additional file 1: Figure S2A and S2B). In co-culture of the adipocyte differentiated from hPreAd, the migration of DCIS.com and MDA-MB-231 cells was significantly increased, while the proliferation of DCIS.com was suppressed (Additional file 1: Figure S3A and S3B).

### IL-6/IL-6R mediated cross-talk between DCIS.com and preadipocytes is associated with the increased proliferation and migration of DCIS.com

**Fig. 3:**
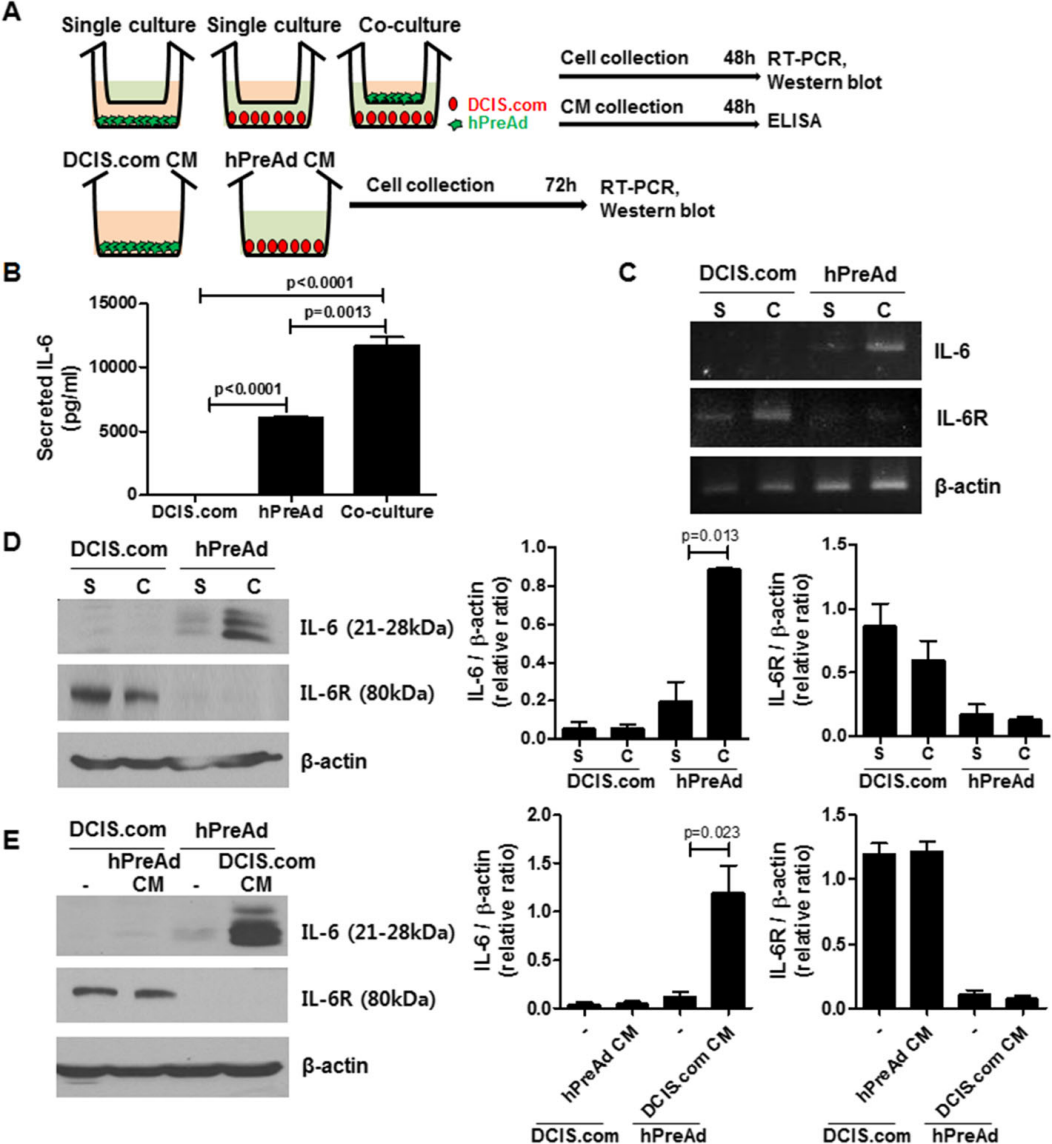
IL-6 expression in human preadipocytes was increased by co-culture with human breast DCIS cells. **a** Experimental scheme illustrating the transwell culture of DCIS.com (DCIS.com) and human preadipocytes (hPreAd). **b** Measurement of secreted IL-6 expression in single or co-culture, as assessed by ELISA. Data are presented as the means ± standard errors of three independent experiments. **c** Analysis of IL-6 and IL-6 receptor (IL-6R) mRNA expression levels in DCIS.com and hPreAd in single culture (S) or co-culture (C), as assessed by RT-PCR. **d** Analysis of IL-6 and IL-6R protein expression levels in DCIS.com and hPreAd in single culture (S) or co-culture (C), as assessed by western blot. **e** Analysis of IL-6 and IL-6R protein expression levels in DCIS.com and hPreAd treated with each conditioned medium (CM), as assessed by western blot

To identify the major factors secreted from hPreAd that contribute to the proliferation migration, and invasion of DCIS.com as shown in experimental scheme (Fig. 3a), we analyzed the adipokines and cytokines in conditioned media after 48 h of single or co-culture as indicated in Table 1. Among the detected adipokines and cytokines, the levels of adiponectin, MMP-9, and IL-2 secreted from DCIS.com were higher than those of hPreAd, but the levels of adipsin, PAI-1, CCL2, CRP, MMP-1, MMP-2, MMP-3 and IL-6 secreted from hPreAd were higher than those of DCIS.com. The other cytokines and adipokines were either rarely detected or not detected. Intriguingly, IL-6 expression levels (11,683 ± 678.3 pg/ml) were approximately 2-fold higher in conditioned media from co-culture than those from the hPreAd single culture (6066 ± 138.7 pg/ml) (Fig. 3b, *P* = 0.0013). To further investigate the change in IL-6 and IL-6 receptor mRNA and protein expression levels in DCIS.com and hPreAd after co-culture or treatment with conditioned media, RT-PCR and western blotting were performed. IL-6 mRNA and protein expression levels were detected in hPreAd, and IL-6R mRNA and protein expression levels were detected in DCIS.com. A significant increase in the mRNA and protein expression levels of IL-6 was observed in hPreAd in co-culture and after treatment with conditioned media compared to those in the single culture, and the mRNA and protein expression levels of IL-6R were not altered in DCIS.com in co-culture or after treatment with conditioned media (Fig. 3c, d, and e).

**Table 1:** Cytokine and adipokine levels in conditioned medium of single or co-cultured human preadipocytes and MCF10DCIS.com cells

	MCF10DCIS.com (pg/ml)	Preadipocyte (pg/ml)	Co-culture (pg/ml)
Adiponectin	116.5 ± 27.6	22.69 ± 13.6	74.2 ± 29.4
Leptin	OOR<	OOR<	OOR<
Adipsin	2.2 ± 0.0	1236.0 ± 128.5	1129.0 ± 158.0
Resistin	44.6 ± 6.63	42.4 ± 4.9	43.8 ± 4.2
PAI-1	1069.0 ± 55.9	17,568.0 ± 1213.0	16,371.0 ± 1418.0
CCL2	1.3 ± 0.1	1825.0 ± 149.0	2160.0 ± 68.4
CRP	0.22 ± 0.0	2.53 ± 0.0	2.53 ± 0.0
TGF-β1	OOR<	OOR<	OOR<
TGF-β2	OOR<	OOR<	OOR<
TGF-β3	OOR<	OOR<	OOR<
IL-2	2.30 ± 0.3	OOR<	OOR<
IL-4	0.26 ± 0.05	0.17 ± 0.0	0.17 ± 0.03
IL-5	0.26 ± 0.05	0.15 ± 0.02	0.17 ± 0.03
IL-6	20.8 ± 0.4	6066.0 ± 138.7	11,683.0 ± 678.3
IL-10	1.14 ± 0.06	0.77 ± 0.04	0.78 ± 0.04
IL-12(p70)	0.94 ± 0.03	0.81 ± 0.06	0.90 ± 0.03
IL-13	0.24 ± 0.06	0.24 ± 0.06	0.24 ± 0.0
GM-CSF	0.60 ± 0.27	OOR<	OOR < −
INF-γ	OOR<	0.91 ± 0.18	OOR<
TNF-α	1.93 ± 0.17	1.59 ± 0.13	1.82 ± 0.13
MMP-1	29.5 ± 1.6	908.6 ± 11.1	787.7 ± 18.9
MMP-2	360.0 ± 11.2	32,069.0 ± 6545	7284.0 ± 435.4
MMP-3	OOR<	665.8 ± 14.1	640.0 ± 32.5
MMP-9	775.7 ± 39.6	199.6 ± 3.6	192.3 ± 14.3
MMP-12	0.16 ± 0.16	OOR<	OOR<
MMP-13	OOR<	OOR<	OOR<

OOR<, out of range below

**Fig. 4:**
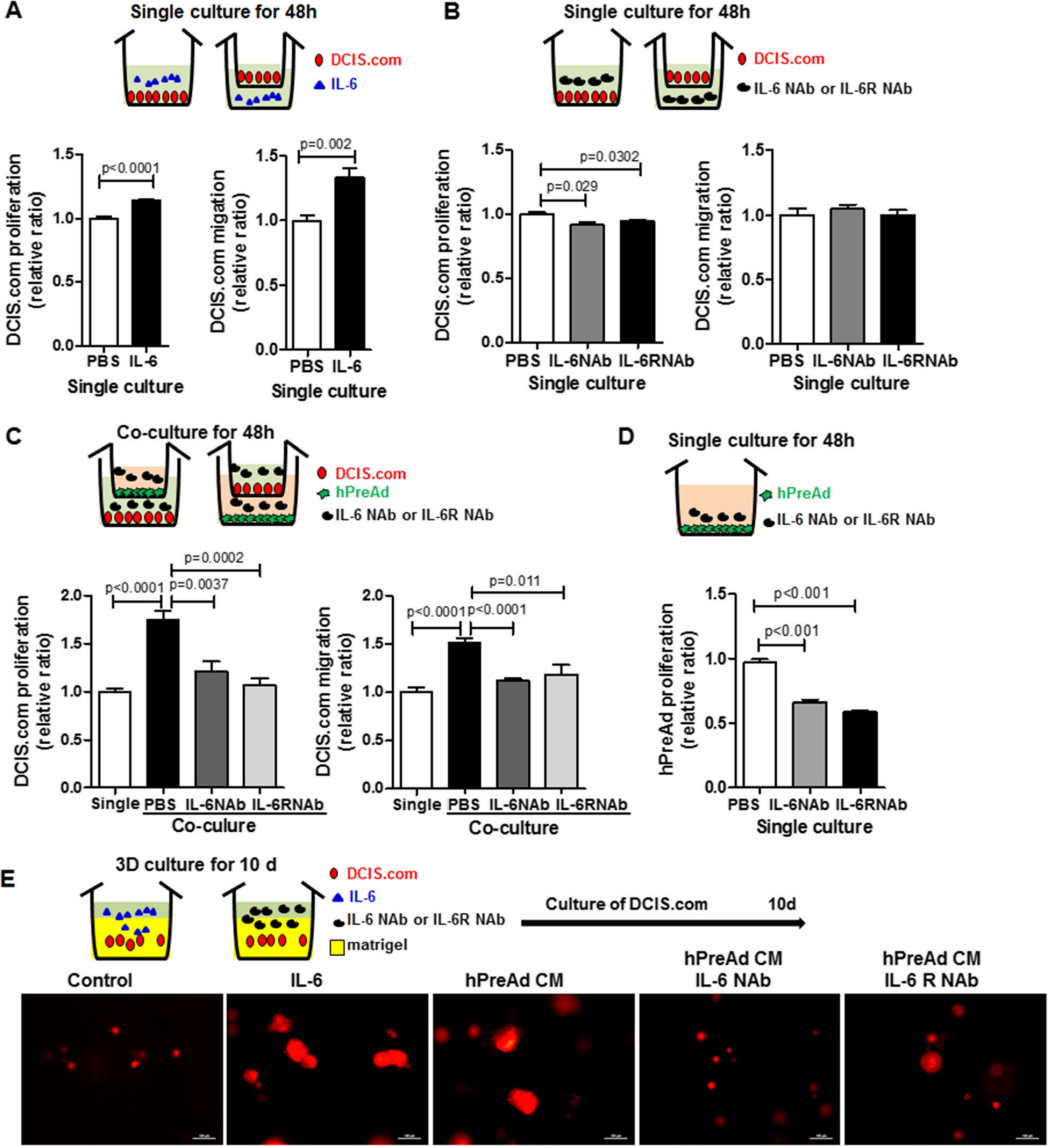
Blocking IL-6 signaling with IL-6 neutralizing antibody or IL-6 receptor antibody abrogates the DCIS.com migration and proliferation enhanced by co-culture with human preadipocytes. Experimental scheme illustrating the transwell culture and 3D culture of DCIS.com (DCIS.com) and human preadipocytes (hPreAd). **a** Proliferation and migration analysis of DCIS.com treated with IL-6 (50 ng/ml), as assessed by MTT assay of six independent experiments. **b** Proliferation and migration analysis of DCIS.com in the presence or absence of IL-6 neutralizing antibody (IL-6 NAb, 3 μg/ml) or IL-6 receptor antibody (IL-6Rα NAb, 3 μg/ml), as assessed by crystal violet assay of three independent experiments. **c** Proliferation and migration analysis of DCIS.com in co-culture in the presence or absence of IL-6 neutralizing antibody (IL-6 NAb, 3 μg/ml) or IL-6 receptor antibody (IL-6Rα NAb, 3 μg/ml), as assessed by crystal violet assay of six independent experiments. **d** Proliferation analysis of hPreAd in the presence or absence of IL-6 neutralizing antibody (IL-6 NAb, 3 μg/ml) or IL-6 receptor antibody (IL-6Rα NAb, 3 μg/ml), as assessed by crystal violet assay of six independent experiments. **e** Fluorescence microscopy images of DCIS.com spheroids in 3D Matrigel culture in the presence or absence of with IL-6 (50 ng/ml), IL-6 NAb (3 μg/ml) or IL-6R NAb (3 μg/ml). All data are presented as the means ± standard errors

Next, we investigated whether the IL-6-mediated cross-talk between DCIS.com and hPreAd was involved in breast cancer cell migration and proliferation. After IL-6 (50 ng/ml) was added to the culture medium of DCIS.com alone for 48 h, DCIS.com proliferation (1.14 ± 0.01, *P* < 0.0001) and migration (1.33 ± 0.07, *P* = 0.002) were significantly increased compared to those of the control cells (Fig. 4a). The treatment with IL-6 (50 ng/ml) for 24 h–72 h promoted migration of MCF10A but not SUM225 cells (Additional file 1: Figure S4). After blocking IL-6 and IL-6R with neutralizing antibodies in the single culture and co-culture, the proliferation and migration of DCIS.com were evaluated using transwell assays. The treatment with 3 μg/ml IL-6NAb or IL-6RNAb in the single culture resulted in a significant decrease of DCIS.com proliferation (0.92 ± 0.02, *P* = 0.03 and 0.92 ± 0.02, *P* = 0.03) but not DCIS.com migration (Fig. 4b). DCIS.com proliferation (1.75 ± 0.09, *P* < 0.0001) promoted by co-culture reverted to a single culture state in the presence of 3 μg/ml IL-6 NAb or IL-6R NAb (1.21 ± 0.10, *P* = 0.0037 or 1.06 ± 0.07, *P* = 0.0002), and migration (1.52 ± 0.04, *P* < 0.0001) enhanced by co-culture was also significantly decreased in IL-6 NAb- or IL-6R NAb-treated DCIS.com (1.11 ± 0.02, *P* < 0.0001 or 1.18 ± 0.09, *P* = 0.011) (Fig. 4c). The hPreAd proliferation ability was significantly suppressed by IL-6 NAbs (0.66 ± 0.02, *P* < 0.0001) and IL-6R NAbs (0.59 ± 0.01, *P* < 0.0001) compared to that of the control cells (Fig. 4d). The effects of IL-6, IL-6 NAb or IL-6R NAb treatment on spheroid colonies in a 3D Matrigel-based culture system were evaluated. The spheroids were usually larger in DCIS.com cultures treated with 50 ng/ml IL-6 or with hPreAd conditioned media than in the untreated control, but administration of 3 μg/ml IL-6 NAb or IL-6R NAb caused the decrease in the spheroid diameter which had been increased by hPreAd conditioned media (Fig. 4e).

We also investigated the phosphorylation of IL-6-triggered signaling molecules in DCIS.com after the administration of hPreAd conditioned media. The phosphorylation levels of mTOR, FAK, AKT, and ERK1/2 were increased in a time-dependent manner by the administration of hPreAd conditioned media (Additional file 1: Figure S5).

### Administration of IL-6 NAb suppressed tumor growth in a mouse xenograft model

**Fig. 5:**
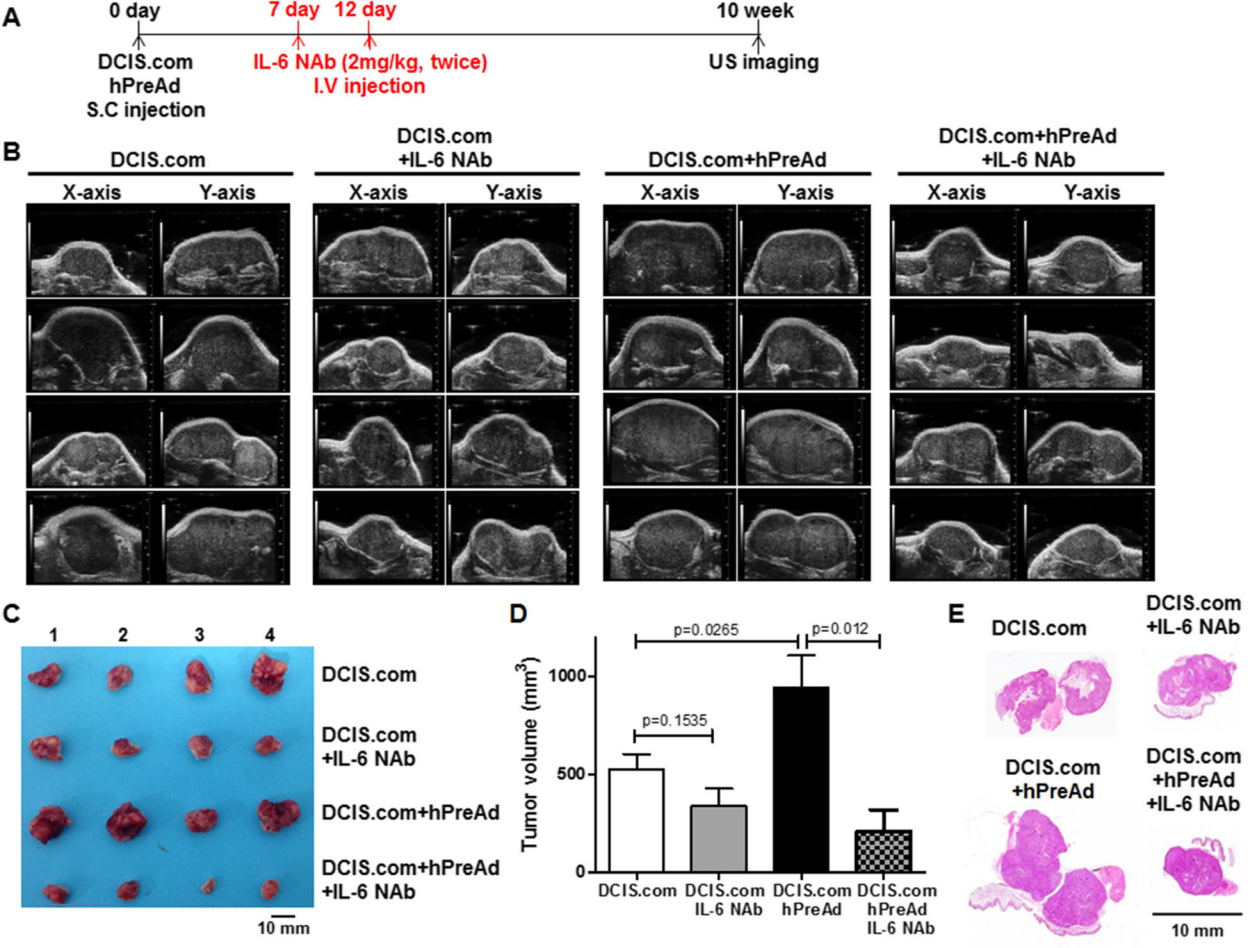
Xenograft tumor volumes in mice were decreased by peritoneal injections of IL-6 neutralizing antibody. **a** In vivo experimental design and treatment schedule with the IL-6 neutralizing antibody (IL-6 NAb, 2 mg/kg). **b** Ultrasound images of xenograft tumors from four mice in each experimental group at 10 weeks post-injection and after two treatments of IL-6 NAb. Two orthogonal images in the X- and Y-axis were obtained. **c** Gross images of the tumors removed from four mice in each group. Bar = 10 mm. **d** Tumor volumes (means ± standard errors) measured from four mice in each group. **e** Representative H&E images of whole tumor tissue slides removed from mice in each group

We finally explored the IL-6-mediated cross-talk between DCIS.com and hPreAd in a mouse xenograft model. We observed the areas of carcinoma invading into the adjacent adipose tissue in the xenograft tumors obtained by co-injection of DCIS.com and preadipocytes (Additional file 1: Figure S6A). Immunostaining of IL-6 revealed that a significant staining for IL-6 was found in fibroblast-like cells located at peritumoral area not adipocytes (Additional file 1: Figure S6B). Fig. 5a shows the schedule for evaluating the IL-6 NAb therapeutic effect in vivo. Using this regimen, two treatments of IL-6 NAb (2 mg/kg) did not cause significant changes in the body weights of mice compared with those of untreated mice, suggesting the absence of physical distress over the course of the experiment. To investigate the in vivo therapeutic efficacy of IL-6 NAb in tumors, we measured tumor volumes using US imaging. Fig. 5b shows the US imaging of tumors in each group at 10 weeks. As shown in Fig. 5c and d, the tumor volumes were significantly increased in the mice co-injected with DCIS.com and hPreAd (942.8 ± 163.0 mm^3^) relative to the mice injected with DCIS.com alone (527.1 ± 77.6 mm^3^, *P* = 0.0.027). The volume of tumors treated with IL-6 NAb was decreased in the mice co-injected with DCIS.com and hPreAd (207.8 ± 111.5 mm^3^, *P* = 0.012) or in the mice injected with DCIS.com alone (338.1 ± 89.4, *P* = 0.154) relative to the untreated mice. Progression of solid DCIS into comedo-DCIS is initiated by naturally occurring apoptosis [15]. In H&E analysis of whole tumor tissue slides a more invasive phenotype and large necrotic areas was observed in hPreAd co-injected tumors relative to the other tumors at 8–10 weeks post-injection, and the treatment with IL-6 NAb inhibited tumor growth and progression of DCIS.com in subcutaneous xenografts (Fig. 5e), indicating that hPreAd promotes tumor growth and progression of DCIS.com via IL-6-mediated signaling.

## Discussion

The importance of the tumor stromal cells surrounding the DCIS in breast cancer progression has been recognized. The role of fibroblast-like preadipocytes in breast tumor progression at early stages is largely unknown. Therefore, in this study, we explored the association of preadipocytes with early stage breast tumor progression. We demonstrated that IL-6 secreted from preadipocytes is a crucial molecule in the cross-talk between preadipocytes and DCIS cells in the early stage of breast cancer development. In this study, we used DCIS.com cells, which are derived from the non-cancerous breast epithelial cell line MCF10A and compose the only human breast cancer cell line that forms DCIS xenografts [16, 17], and preadipocytes, which are isolated from human subcutaneous adipose tissue. After co-culturing with preadipocytes, DCIS.com proliferation and migration were significantly promoted and the differentiation ability of preadipcytes into mature adipocytes, as evidenced by the reduction in lipid droplet formation and the expression of adipocyte-related genes (PPAR-γ and LPL) was inhibited by DCIS.com. IL-6 secretion was greatly increased by co-culture with DCIS.com. Blocking the IL-6-mediated cross-talk with neutralizing antibodies for IL-6 or IL-6R inhibited the DCIS.com proliferation and migration that was enhanced by co-culture with preadipocytes. In the mouse xenograft model, tumor growth and the invasion of primary tumor cells into stroma were promoted by co-injection with preadipocytes. Blocking IL-6 signaling by the intravenous injection of IL-6 neutralizing antibody resulted in suppressed tumor growth compared with that of the controls, which was monitored in vivo with US imaging. Our study represents, to our knowledge, the first trial to examine the impact of preadipocytes and IL-6 in the use of DCIS cells as a breast cancer model of early stage. We have demonstrated that the interaction between human preadipocytes and breast DCIS cells significantly changed the biological behavior of both preadipocytes and breast carcinoma cells.

The role of the microenvironment in the regulation of normal mammary gland function and the microenvironmental changes that occur in breast cancer have gained attention [18]. The most abundant stromal component in the breast is adipose tissue. In breast adipose tissue, preadipocytes with a fibroblastic appearance become mature adipocytes and maintain multipotency, readily differentiating into fibrogenic, osteogenic, and chondrogenic lineages in vitro [19]. To date, very little is known regarding the role of preadipocytes in mammary carcinogenesis. A recent study found that preadipocyte-derived exosomes enhance in vitro cell migration and the self-renewal of DCIS cells and facilitated the xenograft tumor formation of DCIS cells in vivo, suggesting that a number of cytokines within preadipocyte-secreted exosomes might contribute to DCIS tumor progression [14]. Likewise, we found that co-culture with human preadipocytes or the conditioned media of human preadipocytes promoted the in vitro migration and proliferation of MCF10DCIS.com (basal-like DCIS model) but not SUM225 (HER-2+/luminal DCIS model). The studies by Dirat B et al. and Manabe Y et al. [20, 21] showed the preadipocytes do not enhance the migration, invasion or proliferation of ZR 75.1, MCF-7, and T47D (luminal-like subtype) in contrast to SUM159PT (basal-like subtype). Their results are similar to our data obtained from SUM225 (HER-2+/luminal-like subtype) and DCIS.com and MDA-MB-231 (basal-like subtype). Likewise, adipocytes differentiated from preadipocytes remarkably enhanced the migration of DCIS.com and MDA-231, but not SUM225. Taken together, the impact of preadipocytes on the proliferation and migration of breast tumor cells appears to the difference between basal-like and luminal-like subtypes. Our results highlight that preadipocytes differentially act on tumor cell proliferation and migration depending on the intrinsic or molecular subtypes of breast tumor cells and are able to more effectively stimulate the proliferative and migratory capacities of basal-like breast cancer cells. To evaluate this similarity and difference in more detail, further studies regarding the production of cytokines and adipokines in culture system are needed.

According to study reported by Bochet L et al. [22], SUM159PT cells force mature adipocytes towards fibroblast-like cells exhibiting elongated morphology and expressing FSP1, but not α-SMA in adipose tissue, and acquire more aggressive and invasive capability of tumor cells. Similarly, our study demonstrated that DCIS.com cells suppressed the differentiation of preadipocyte into mature adipocyte, and made preadipocytes have more elongated fibroblast morphology accompanying with the upregulation of FSP1 and α-SMC, resulting in the acquisition of aggressive phenotype with a high proliferative and migratory activity. These data, taken together, preadipocytes and adipocyte-derived fibroblasts are morphologically and phenotypically similar to fibroblast-like cells and have cooperative roles in promoting breast cancer progression. The further study to analyze gene expression and signaling molecules in preadipocytes and adipocyte-derived fibroblasts from co-culture with breast cancer cells may provide large amounts of additional information to define their similarities and differences of biological functions. Taken together with such observations, we suggest that stromal cells including preadipocytes and adipocyte-derived fibroblasts in the breast tumor microenvironment represent attractive targets for anti-cancer therapeutics.

Adipocytes are known to play a significant role in promoting tumor progression by secreting cytokines including IL-6, IL-1, IL-8, and TNF-α [20]. Adipose tissue is an important source of circulating IL-6 [23]. IL-6 stimulates lipolysis, and also inhibits adipose tissue lipoprotein lipase activity in human adipose breast tissue [24]. IL-6 is secreted by human adipose stromal cells which are isolated from abdominal and breast adipose tissue and also referred to as preadipocytes [7]. Expression of IL-6 is greater in mouse preadipocytes (3 T3-L1 cell) than in differentiated adipocytes, suggesting that preadipocytes are the source of adipose tissue IL-6 [25]. Similarly, we here found a significant staining for IL-6 in fibroblast-like cells located at peritumoral area not adipocytes. Furthermore, tumor tissues co-injected with DCIS.com and preadipocytes displayed stronger IL-6 staining than those injected with DCIS.com alone. These results imply that IL-6 positive fibroblast-like cells which can be derived from preadipocytes and adipocytes may play an important role in DCIS tumor growth and invasive progression.

IL-6 is an autocrine and paracrine regulator in cell proliferation, survival, and metabolism, but has lesser actions on adipose tissue [23]. IL-6/IL-6 receptor mediated signaling leads to activation of the JAK family of tyrosine kinases, which then stimulate multiple pathways involving MAPKs, PI3Ks, STATs, and other signaling proteins [26]. IL-6-mediated signaling modulates cell migration and the epithelial to mesenchymal transition of ER-positive breast DCIS cells ZR-75-1 via the down-regulation of E-cadherin [27]. A recent study showed that the IL-6 signaling between human breast DCIS cells (DCIS.com and SUM102) and stromal fibroblasts represents an important factor in the initiation of DCIS cell progression to invasive breast carcinoma [28]. In the present study, we observed high IL-6 expression in human preadipocytes co-cultured with DCIS.com as well as increased phosphorylation of mTOR, AKT, ERK and STAT3 in DCIS.com treated with conditioned media of human preadipocytes. Moreover, IL-6 treatment caused a significant increase in DCIS.com cell proliferation and migration, but no additional migration effect of IL-6 on SUM225 was observed, indicating that the sensitivity to IL-6 is absent in SUM225 or the different factors involved in the reciprocal cross talk between preadipocytes and SUM225 may not exert significant effects on migration of SUM225 cells. Our data, together with our findings that implicate IL-6 secreted by preadipocytes in breast cancer progression from basal-like DCIS, strongly support a working hypothesis that resident preadipocytes in local adipose stromal tissues and their secretory factor IL-6 play a key role in mediating the progression of early stage breast cancer.

We aimed to identify the most important soluble factor secreted from human preadipocytes that promotes the invasion ability of DCIS.com and tumor growth in the reciprocal communication between the preadipocytes and DCIS.com. After extensively examining the changes in cytokines and adipokines in co-culture medium, we found a significant upregulation in IL-6 expression. Co-culture with hPreAd and treatment with IL-6 promoted the migration and proliferation ability of DCIS.com, whereas treatment with IL-6 NAb or IL-6R NAb abrogated the DCIS.com migration and proliferation enhanced by co-culture with hPreAd. Our current results are in accordance with previously published studies [14, 27] and indicate that IL-6/IL-6R mediated signaling is a critical therapeutic target for inhibiting early stage breast tumor progression.

Clinical studies have revealed that a high serum expression level of IL-6 in patients is associated with advanced tumor stages in various cancers (multiple myeloma, non-small cell lung carcinoma, colorectal cancer, renal cell carcinoma, prostate cancer, breast cancer and ovarian cancer) [29–32]. Moreover, IL-6 is associated with a poor prognosis in breast cancer patients [33]. Therefore, blocking IL-6 signaling is a potential therapeutic strategy for cancer patients. The IL-6 antibody (siltuximab, CNTO 328) and IL-6 receptor antibody (tocilizumab) belong to a class of therapeutics clinically approved for patients with malignant cancers, including prostate, ovarian, and lung cancers [29–32]. In our study, notably, blocking IL-6 signaling with intravenous injections of IL-6 neutralizing antibodies suppressed xenograft tumor growth, suggesting that IL-6 signaling would be therapeutic target for inhibiting early stage breast tumor progression.

## Conclusions

In summary, we propose that IL-6 is an important mediator involved in the cross-talk between preadipocytes and breast DCIS cells, which is associated with early stage breast cancer progression. Therefore, blocking IL-6 signaling might be a potential therapeutic strategy for breast DCIS characterized by pathological IL-6 overproduction. Deciphering the critical factor behind preadipocyte-breast DCIS cell cross-talk might provide new targets for improving breast cancer diagnosis and for the design of innovative therapeutic strategies.

## Additional file

Additional file 1: Supplementary Figures and Table. (DOCX 6073 kb)

## Abbreviations

3D: Three-dimensional
CCL5: C-c motif chemokine 5
DCIS: Ductal carcinoma in situ
DMEM: Dulbecco’s Modified Eagle Medium
H&E: Hematoxylin and eosin
hPreAd: Human preadipocyte
IL-6: Interleukin-6
NAb: Neutralizing antibody
RT-PCR: Real-time PCR
