# Supplementary material for: IL-6-mediated cross-talk between human preadipocytes and ductal carcinoma in situ in breast cancer progression
Source: J Exp Clin Cancer Res. 2018 Aug 22;37:200. doi: 10.1186/s13046-018-0867-3 (PMC6106749; doi:10.1186/s13046-018-0867-3)
Supplement: Supplementary file 1 — Supplementary Figures and Table. (DOCX 6073 kb) [file 13046_2018_867_MOESM1_ESM.docx]

**Supplementary Materials**

**IL-6-mediated cross-talk between human preadipocytes and duc**tal carcinoma *in situ* in breast cancer progression

Hoe Suk Kim^1^, Minji Jung^1^, Sul Ki Choi^1,2^, Jisu Woo^1^, Yin Ji Piao^1,2^, Eun Hye Hwang^1^, Hyelim Kim^1,2^, Seung Ja Kim^3^, Woo Kyung Moon^1,2,*^

**Correspondence to:** Woo Kyung Moon, e-mail: [moonwk@snu.ac.kr](mailto:moonwk@snu.ac.kr)


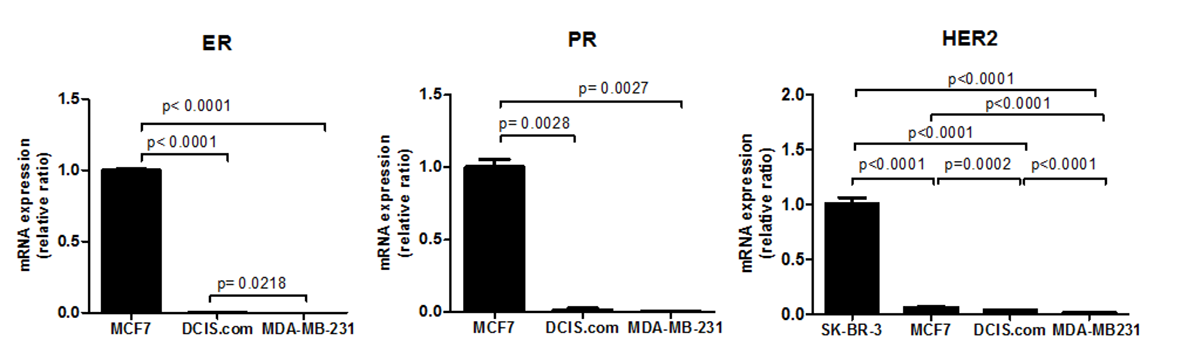


**Fig. S1** MCF10DCIS.com cells do not express estrogen receptor (ER), progesterone receptor (PR) and human epithelial receptor 2 (HER2). ER, PR and HER2 mRNA expressions in MCF-7, MCF10DCIS.com, SK-BR-3, and MDA-MB-231 cells, as assessed by real-time RT-PCR (n=3).

**
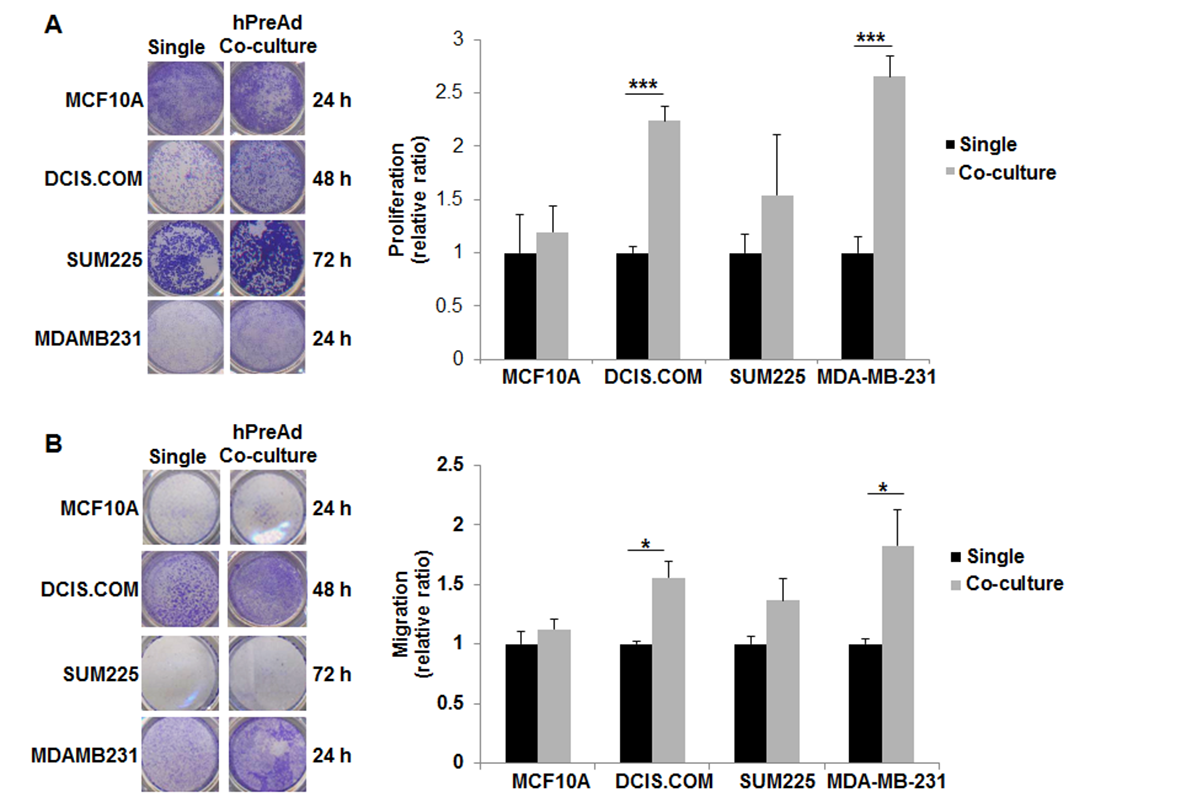
**

**Fig. S2** Analysis of the proliferation and migration abilities of MCF10A, MCF10DCIS.com, SUM225, and MDA-MB-231 cells in co-culture with human preadipocytes. **(a)** Proliferation (n=3) and **(b)** migration analysis (n=6) of each cell in single or co-culture with human preadipocyte (hPreAd), as assessed by crystal violet assay. All data are presented as the means ± standard deviation. ** p<0.01


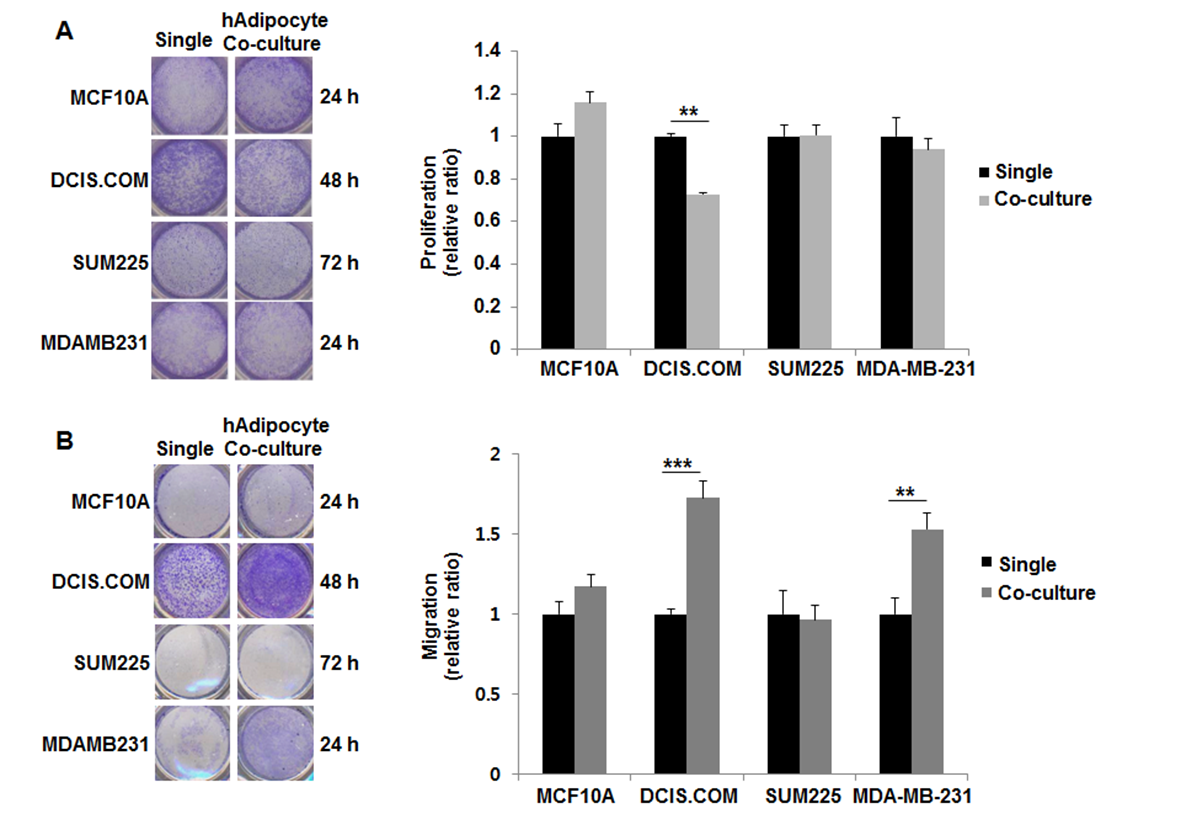


**Fig. S3** Analysis of the proliferation and migration abilities of MCF10A, MCF10DCIS.com, SUM225, and MDA-MB-231 cells in co-culture with adipocyte differentiated from human preadipocytes. **(a)** Proliferation (n=3) and **(b)** migration (n=3) analysis of each cell in single or co-culture with human adipocyte (hAdipocyte), as assessed by crystal violet assay. All data are presented as the means ± standard deviations. * p<0.05, ** p<0.01


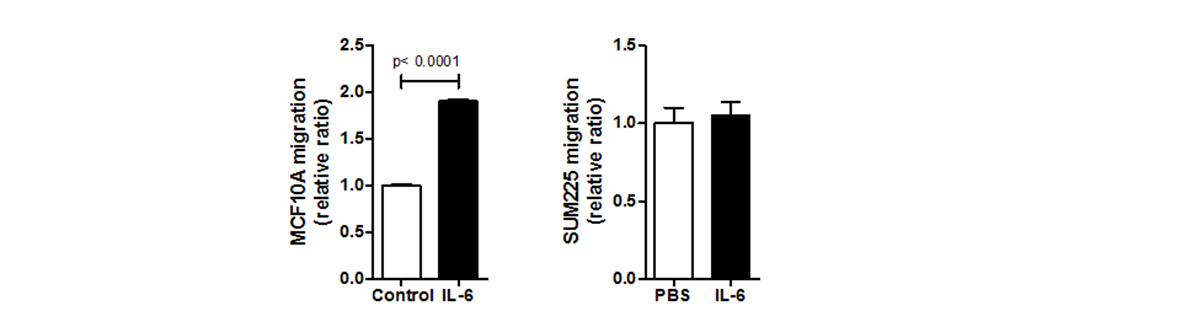


**Fig. S4** Proliferation and migration analysis of MCF10A and SUM225 treated with IL-6 (50 ng/ml), as assessed by crystal violet assay of three independent experiments.


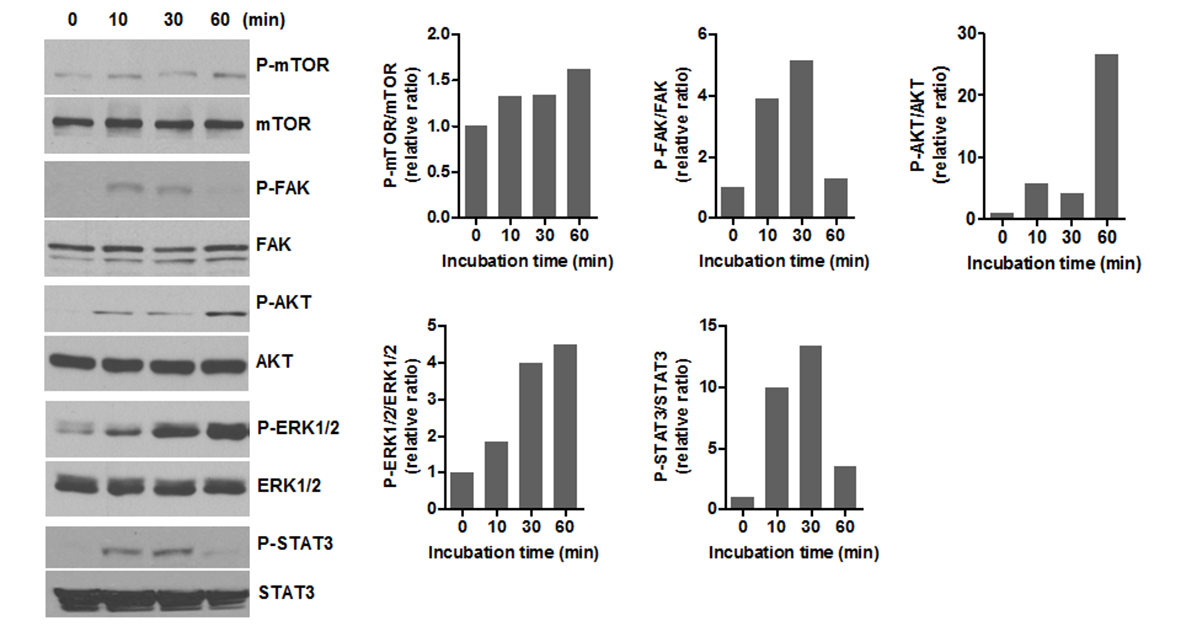


**Fig. S5** Representative western blots of phosphorylated and total mTOR, FAK, AKT, ERK1/2, and STAT3 in MCF10DCIS.com cells treated with the conditioned medium of human preadipocytes.


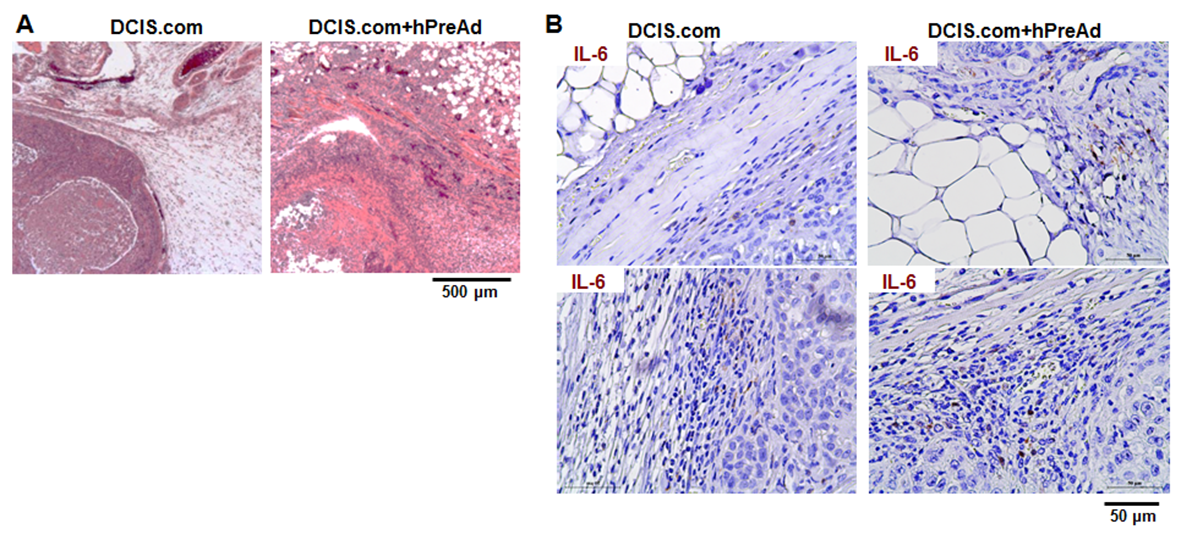


**Fig. S6** Histological analysis of xenograft tumor tissues. **(a)** H&E staining and **(b)** immunostainig of interleukin-6 (IL-6) in tumors injected with DCIS.com or co-injected with DCIS.com and PreAd.

Table S1. Specific primer sequence for RT-PCR and real-time RT-PCR

| **Gene** |  | **Sequence (5′**$\boldsymbol{->}$**3′)** |
| --- | --- | --- |
| IL-6 | Forward | ATGAACTCCTTCTCCACAAGCGC |
|  | Reverse | GAAGAGCCCTCAGGCTGGACTG |
| IL-6R | Forward | CATTGCCATTGTTCTGAGGTTC |
|  | Reverse | AGTAGTCTGTATTGCTGATGTC |
| β-actin | Forward | TTCCTGGGCATGGAGTCCTGTGG |
|  | Reverse | CGCCTAGAAGCATTTGCGGTGG |
| LPL | Forward | ACAAGAGAGAACCAGACTCCAA |
|  | Reverse | AGGGTAGTTAAACTCCTCCTCC |
| PPAR-γ | Forward | GTGGCCGCAGAAATGACC |
|  | Reverse | CCACGGAGCTGATCCCAA |
| ER | Forward | CGCAAATGCTACGAAGTGGG |
|  | Reverse | GGTTGGCAGCTCTCATGTCT |
| PR | Forward | TGACTGAGCTGAAGGCAAAGG |
|  | Reverse | AGGGAGATAGGTATGGCCGAA |
| HER2 | Forward | TGTGAGGCTTCGAAGCTGCA |
|  | Reverse | GGCACCCAGCTCTTTGAGGA |
| β-actin | Forward | TTCCTGGGCATGGAGTCCTG |
|  | Reverse | CGCCTAGAAGCATTTGCGGT |
